# Clinical characteristics and real-life diagnostic approaches in all Danish children with hereditary angioedema

**DOI:** 10.1186/s13023-017-0604-6

**Published:** 2017-03-16

**Authors:** Anne Aabom, Klaus E. Andersen, Christina Fagerberg, Niels Fisker, Marianne A. Jakobsen, Anette Bygum

**Affiliations:** 1 Department of Dermatology and Allergy Centre, Odense University Hospital, Odense, Denmark; 2 OPEN, Odense Patient data Explorative Network, Odense University Hospital, Odense, Denmark; 3 Center for Innovative Medical Technology, Institute for Clinical Research, University of Southern Denmark, Odense, Denmark; 4 Department of Clinical Genetics, Odense University Hospital, Odense, Denmark; 5 Hans Christian Andersen Children’s Hospital, Odense University Hospital, Odense, Denmark; 6 Department of Clinical Immunology, Odense University Hospital, Odense, Denmark

**Keywords:** Clinical characteristics, Complement C4, Diagnosis, Genetic testing, Hereditary angioedema, Pediatric, Therapy

## Abstract

**Background:**

With a potentially early onset, hereditary angioedema (HAE) requires special knowledge also in infancy and early childhood. In children from families with HAE, the diagnosis should be confirmed or refuted early, which can be difficult. Studies of childhood HAE and the diagnostic approaches are limited. Our aim was to investigate the entire Danish cohort of children with HAE and non-HAE children of HAE patients for diagnostic approaches and clinical characteristics.

**Results:**

We included 41 children: 22 with HAE and 19 non-HAE. Of the HAE children, 14 were symptomatic—median age at onset was 4 [1–11] years. The first attack was peripheral in 8/14 children and abdominal in 6/14 children, i.e. no one had their first attacks in the upper airways. Most children had less than one attack per month. All of the symptomatic children had been treated with tranexamic acid and/or C1 inhibitor concentrate. Unlike in other countries, androgens were not used in our pediatric cohort. Home therapy with C1 inhibitor concentrate was established in 9 cases: 6 children were trained in self-administration and 3 children were treated by parents. Of the children, 10 had been diagnosed by symptoms, including 3 without family history—median age of diagnosis among these children was 5.35 [2–13.2] years. In 31 children, HAE was diagnosed or refuted before symptoms by blood samples. In 23 of these children, complement values were investigated, and in 9 cases genetic testing was added to the complement measurements. In 8 children recently investigated, genetic testing was first choice. Cord blood was used for complement measurements in 9 children and for genetic testing in 4 children. Results of complement measurements were equivocal in several cases, especially in the cord blood samples, and the sensitivity of low complement C4 for the diagnosis of HAE was 75%.

**Conclusions:**

We investigated clinical characteristics in all Danish children with HAE. The rate of home therapy was high and androgens had been avoided. Complement values were often equivocal, especially in cord blood samples. Consequently, we have changed diagnostic practice to early genetic testing in children where the family mutation is known.

## Background

Hereditary angioedema (HAE) is a genetic disease characterized by recurrent attacks of subcutaneous swellings and abdominal pain. It is caused by either a deficiency (HAE type I) or a reduced functional capacity (HAE type II) of complement C1 inhibitor. The age of onset is variable, but half the patients develop symptoms before the age of 10 years [1, 2]. Life-threatening attacks may occur in the upper airways, and death due to asphyxiation has been reported in children as young as 2 weeks old [3].

The course of HAE is unpredictable, and it is important to establish a diagnosis early, ideally before onset in cases with a family history. The diagnosis is based on clinical manifestations and/or family history combined with laboratory findings [4].

### Diagnosis based on biochemical measurements

Biochemical investigation comprises the complement values total antigenic C1 inhibitor (C1INH), functional capacity of C1 inhibitor (fC1INH), and complement C4 (C4). According to diagnostic algorithms, a family history of angioedema should lead to complement measurement [5, 6]. For early diagnosis of newborns from HAE families, cord blood has been used [7]. There is consensus that low complement values obtained on two separate occasions (one after the age of 12 months) are required to diagnose HAE in asymptomatic children [4–6, 8]. However, most complement assays lack validated reference values for children. Furthermore, results may be equivocal despite repeated measurements and must be interpreted with caution.

### Diagnosis based on genetic investigation

Alternatively to complement measurements, mutational screening of the gene encoding C1INH can be used [9]. HAE is caused by mutations in the *SERPING1* gene (OMIM #606860) located on chromosome 11. The inheritance pattern is autosomal dominant, but 25% of cases are *de novo* mutations [10]. If the family mutation is known, children can be diagnosed early with genetic testing performed on peripheral blood or blood from the umbilical cord. Until now, this has not been recommended as first choice [11].

With a potentially early onset, HAE requires special knowledge in both diagnosis and overall management, also in infancy and early childhood. However, original studies of the special aspects of childhood HAE, including the diagnostic challenges, are limited. The aim of this study was to describe clinical features of childhood HAE and evaluate the real-life diagnostic approaches in Danish HAE families.

## Methods

This retrospective study was undertaken at the Danish HAE Comprehensive Care Centre, Odense University Hospital in October 2016. Data collection began in January 2013 and children born after 1995 were included. Children were identified via a nationwide HAE registry containing data of all known Danish HAE families [12]. The search had two steps: 1) All children in the registry with a diagnosis of HAE type I or II were identified. For convenience, both symptomatic children and carriers of HAE who had not yet experienced any attacks were all labeled HAE patients. In cases with a family history of HAE, all healthy siblings were included (information about these children was retrieved from medical records of the siblings with HAE or the parents). Half-siblings were also included if they shared the parent with HAE. 2) Medical records of all adult HAE patients that were not parents of children already included were searched for information about other healthy children.

Clinical information from children with HAE (age at onset, localization of attacks, attack frequency, and use of therapy) was retrieved from the medical records. The observation period for attack frequency and use of therapy was 45 months (January 2013–October 2016). During this observation period, children were excluded when they turned 18 years old. Information on the diagnostic process was retrieved from the medical records.

Results of complement measurements and genetic testing were recorded in a research database. C1INH and C4 were measured with nephelometry, turbidimetry, or rocket immunoelectrophoresis and fC1INH with chromogenic assays. Mostly, the analyses were performed by Unilabs, Copenhagen, but in some cases they were performed by other laboratories in Denmark or abroad. In each case, the complement results were interpreted against the performing laboratories’ reference range for healthy adults. On this background, complement values were categorized as “normal” (values within or above adult normal range), “low” (values below adult normal range), or “equivocal” (cases with inconsistency, e.g. low C1INH combined with normal C4). Genetic testing for mutations in the *SERPING1* gene were performed with Sanger sequencing or Multiplex Ligation-dependent Probe Amplification (MLPA) analysis. Parental written consent to investigate the medical records and to contact previous healthcare professionals was granted, and the study was approved by the Danish Data Protection Agency (14/38996).

## Results

In 18 of 35 Danish HAE families, we identified 41 children (27 boys, 14 girls) eligible for the study; all agreed to participate. Of these children, 22 had HAE (3 had type II) and 19 had not (named non-HAE). Among the 19 non-HAE children, all had a parent with HAE and 10 also had a sibling with HAE. Among the 22 children with HAE, 14 were symptomatic. Clinical characteristics are outlined in Table 1.

**Table 1:** Clinical characteristics in 14 symptomatic children with hereditary angioedema

Age at onset (years)	Median 4 [1–11]
Localization of first attack	
Abdominal	6/14 (43%)
Peripheral	8/14 (57%)
Upper airway	0
Types of attacks ever	
Abdominal	14/14 (100%)
Peripheral	11/14 (79%)
Upper airway	4/14 (29%)
Number of attacks within the observation period (45 months)^a^	Median 13 [4–121]
Frequency of attacks within the observation period (45 months)	
None	1/14 (7%)
Rare (< one attack per month)	10/14 (72%)
Frequent (≥ one attack per month)	3/14 (21%)

^a^Not including 4 children who turned 18 years old in the observation period

In the observation period, 4 children turned 18 years old and were thereafter excluded from study. Median age at onset was similar in girls and boys, but age at onset ranged more widely among girls (girls: 5 [1–11] years, boys: 4 [3–5.4] years). There was no correlation between age at onset and number of attacks within the observation period (data not shown).

All of the symptomatic children had received treatment (Table 2).

**Table 2:** Use of treatment among 14 children with hereditary angioedema

		Ever	In the observation period (Jan. 2013–Oct. 2016)
Tranexamic acid	On-demand treatment	9/14	4/14
	Short-term prophylaxis^a^	4/14	1/14
	Long-term prophylaxis^b^	4/14	1/14
C1INH concentrate	On-demand treatment	12/14	12/14
	Short-term prophylaxis^a^	5/14	4/14
	Long-term prophylaxis^c^	1/14	1/14
Icatibant	On-demand treatment^c^	1/14	1/14

^a^Indications for short-term prophylaxis were dental treatment, holiday, school camp, birthday, and other family events

^b^Long-term prophylaxis with tranexamic acid was used in 4 children for 0.2–5.6 years between 2006 and 2013

^c^Off-label

Home therapy with C1INH concentrate was established in 9 cases. In 6 cases, the child was trained in self-administration (at the age 10.4–16.4 years), and in 3 cases treatment was administered by parents (established when children were 5.8–12.1 years old).

The results of all diagnostic blood samples for children in the cohort are outlined in Tables 3 and 4. Mostly, C1INH and C4 had been measured with nephelometry or turbidimetry, and fC1INH with chromogenic assays. In one case, C1INH had been measured with rocket immunoelectrophoresis (HAE5). In a few medical records, “normal” complement values were reported, but the exact laboratory data could not be retrieved. These missing values were noted in the study, but were not given any diagnostic weight.

**Table 3:** Results of diagnostic blood samples in 22 children with HAE

ID sex	Born	Age at onset/diagnosis	Cord blood samples [reference values for adults]	Peripheral complement measurements [reference values for adults]			Genetic testing^b^
HAE1 f	1995	5/6 y	n.d.	6 y fC1INH 34 [70–130]%	**8.8 y** **C1INH 0.15 [0.21–0.39]g/L** **fC1INH 55 [70–130]%** **C4 0.13 [0.1–0.4]g/L**	11.6 y C1INH 129 [210–345]mg/L fC1INH 9.2 [17.2–27.4]U/ml C4 120 [157–257]U/mg	11.6 y c.437delT, p.Leu124TrpfsTer2
HAE2 f	1995	5/6.3 y	Complement values reported as normal (missing)	6.3 y fC1INH 33 [70–130]%	9.5 y C1INH 0.05 [0.21–0.39]g/L fC1INH < 20 [70–130]% C4 0.08 [0.1–0.4]g/L		n.d.
HAE3 m	1996	4/4 y	n.d.	2 y fC1INH 34 [70–130]%	**6.8 y** **C1INH 0.05 [0.21–0.39]g/L** **fC1INH 122 [70–130]%** **C4 0.07 [0.1–0.4]g/L**	8.5 y C1INH 0.06 [0.21–0.39]g/L fC1INH 23 [70–130]%	n.d.
HAE4 f	1996	1/2 y	n.d.	2 y fC1INH 22 [70–130]%	9.7 y C1INH 0.06 [0.21–0.39]g/L fC1INH 39 [70–130]% C4 0.07 [0.1–0.4]g/L		9.8 y c.23dupT, X19 in signal peptide
HAE5^a^ f	1999	11/13.2 y	n.d.	13.2 y C1INH <25 [72–153]% fC1INH 27 [70–130]% C4 0.05 [0.16–0.38]g/L	13.5 y C1INH 0.08 [0.21–0.39]g/L fC1INH 46 [70–130]% C4 < 0.06 [0.1–0.4]g/L		13.7 and 15.1 y No *SERPING1* mutation found
HAE6 m	2000	3/3 y	n.d.	2.3 y C1INH 0.09 [0.21–0.39]g/L fC1INH 47 [70–130]% C4 0.2 [0.49–1.66]g/L			n.d.
HAE7^a^ m	2000	4/8.8 y	n.d.	8.8 y C1INH 0.07 [0.21–0.39]g/L fC1INH 32 [70–130]% C4 0.07 [0.1–0.4] g/L			9.8 y c.1030_1503del, deletion of exons 7 and 8
HAE8^a^ f	2000	4/6.8 y	n.d.	6.8 y fC1INH 20 [70–130]% C4 < 0.04 [0.1–0.4]g/L	6.9 y C1INH 0.14 [0.21–0.39]g/L fC1INH 21 [70–130]% C4 < 0.04 [0.1–0.4]g/L		7.5 y c.1381G > C, p.Ala439Pro
HAE9 f	2001	10/1.6 y	**C1INH 0.07 g/L [0.21–0.39]** **fC1INH 32% [70–130]** **C4 0.15 g/L [0.1–0.4]**	1.6 y C1INH 0.12 [0.21–0.39]g/L C4 0.08 [0.1–0.4]g/L	2.8 y fC1INH 49 [70–130]%	7.3 y C1INH 0.13 [0.21–0.39]g/L fC1INH 50 [70–130]% C4 0.08 [0.1–0.4]g/L	n.d.
HAE10 f	2004	4.2/4.7 y	n.d.	4.7 y C1INH 0.06 [0.21–0.39]g/L fC1INH 32 [70–130]%	4.8 y C1INH 61 [210–345]mg/L fC1INH <2 [17.2–27.4]U/ml C4 68 [157–257]mg/L		4.8 y c.551_685del, deletion of exon 4
HAE11 m type II	2004	-/5.7 y	n.d.	5 y C1INH 0.44 [0.21–0.39]g/L fC1INH 56 [70–130]% C4 0.07 [0.1–0.4]g/L	5.7 y C1INH 0.34 [0.21–0.39]g/L fC1INH 37 [70–130]%		6.3 y c.1397G > A, p.Arg444His
HAE12 m	2006	4/4.4 y	C1INH 0.05 [0.21–0.39]g/L C4 0.08 [0.1–0.4]g/L	**4.4 y** **fC1INH 37 [70–130]%** **C4 0.1 [0.1–0.4]g/L**			4.5 y c.1417G > A, p.Val451Met
HAE13 m	2006	5.4/3.8 y	fC1INH 32 [70–130]%	**1.3 y** **fC1INH 69 [70–130]%** **C4 0.11 [0.1–0.4]g/L**	2.6 y C4 0.08 [0.1–0.4]g/L		3.8 y c.668_669delA, p.Gln201fsTer10
HAE14 m	2008	3.3/2.7 y	C1INH <0.03 [0.21–0.39]g/L fC1INH <20 [70–130]% C4 0.05 [0.1–0.4]g/L	**1.1 y** **C1INH <0.25 [0.21–0.39]g/L** **fC1INH 65 [70–130]%** **C4 0.04 [0.1–0.4]g/L**			2.7 y c.1-22-2A > G, splicing defect
HAE15 m	2009	-/5.3 y	n.d.	**2.4 y** **C1INH 0.16 [0.21–0.39]g/L** **fC1INH 65 [70–130]%** **C4 0.2 [0.1–0.4]g/L**	**4.4 y** **C1INH 0.07 [0.21–0.39]g/L** **fC1INH 83 [70–130]%** **C4 0.07 [0.1–0.4]g/L**		5.3 y c.1250-1G > A, splicing defect
HAE16 m	2009	3.8/1 y	C1INH <0.03 [0.21–0.39]g/L C4 0.04 [0.1–0.4]g/L	**1 y** **C1INH 0.13 [0.21–0.39]g/L** **fC1INH 52 [70–130]%** **C4 0.11 [0.1–0.4]g/L**			1.7 y c.551_685del, deletion of exon 4
HAE17 m type II	2012	-/2.8 y	n.d.				2.8 y c.1396 C > T, p.Arg444Cys
HAE18 m	2012	-/2.1 y	n.d.				2.1 y c.762_763delCA, p.Asn232LysfsTer2
HAE19 m	2012	-/1.8 y	fC1INH 20 [70–130]%	0.9 y C1INH 0.10 [0.21–0.39]g/L fC1INH 56 [70–130]% C4 0.08 [0.1–0.4]g/L			1.8 y c.1250-1G > A
HAE20 m	2013	-/0.5 y	**C1INH 0.08 [0.21–0.39]g/L** **C4 0.14 [0.1–0.4]g/L**				0.5 y c.1-22-2A > C, splicing defect
HAE21 f type II	2015	-/0 y	Genetic testing, mutation found				0 y c.1396 C > T, p.Arg444Cys
HAE22 m	2016	-/0 y	Genetic testing, mutation found				0 y c.762_763delCA, p.Asn232LysfsTer2

Complement values were equivocal (bold font) or low compared with adult normal range

*N.d*. not determined. - asymptomatic

^a^sporadic HAE (no family history)

^b^amino acid numbering based on the mature protein (Genbank accession number #NM_000062)

**Table 4:** Results of diagnostic blood samples in 19 non-HAE children

ID sex	Born	Age when HAE was refuted	Cord blood samples [reference values for adults]	Peripheral complement measurements [reference values for adults]		Genetic testing
Non1 m	1996	4.8 y	n.d.	4.8 y fC1INH 121 [70–130]%		n.d.
Non2 m	1997	1.5 y	n.d.	1.5 y C1INH 162 [80–140]units fC1INH 168 [70–130]%		n.d.
Non3 m	1997	18.1 y	n.d.	2.9 y Reported as normal, but missing		18.1 y Family mutation ruled out
Non4 f	1997	14.9 y	n.d.	<14.6 y (exact age unknown) Reported as normal (missing)	14.9 y C1INH 0.33 [0.21–0.39]g/L fC1INH 120 [70–130]% C4 0.18 [0.1–0.4]g/L	n.d.
Non5 m	1999	15.7 y	n.d.	0.1 y fC1INH 81 [70–130]%		15.7 y Family mutation ruled out
Non6 m	1999	16.1 y	n.d	1 y fC1INH 166 [70–130]%		16.1 y Family mutation ruled out
Non7 f	1999	1.9 y	n.d.	1.9 y fC1INH 110 [70–130]%		n.d.
Non8 f	2000	6.4 y	n.d.	6.4 y C1INH 0.27 [0.21–0.39]g/L fC1INH 106 [70–130]% C4 0.2 [0.1–0.4]g/L		n.d.
Non9 m	2000	5.9 y	n.d.	4.3 y C1INH 0.34 [0.21–0.39]g/L fC1INH 129 [70–130]% C4 0.11 [0.1–0.4]g/L	5.9 y C1INH 0.34 [0.21–0.39]g/L fC1INH 135 [70–130]% C4 0.19 [0.1–0.4]g/L	n.d.
Non10 m	2002	9.1 y	n.d.	9.1 y C1INH 0.32 [0.21–0.39]g/L fC1INH 116 [70–130]% C4 0.27 [0.1–0.4]g/L		n.d.
Non11 m	2003	3.2 y	n.d.	1.5 y C1INH 0.38 [0.21–0.39]g/L fC1INH 117 [70–130]% C4 0.13 [0.1–0.4]g/L	3.2 y C1INH 0.32 [0.21–0.39] g/L fC1INH 134 [70–130]% C4 0.22 [0.1–0.4]g/L	n.d.
Non12 m	2008	1.3 y	n.d.	1.3 y C1INH 0.4 [0.21–0.39]g/L fC1INH 174 [70–130]% C4 0.33 [0.1–0.4]g/L		n.d.
Non13 m	2008	1 y	Taken, but not analyzed	0.8 y C1INH 0.41 [0.21–0.39]g/L fC1INH 179 [70–130]% C4 0.31 [0.1–0.4]g/L	1 y C1INH 0.35 [0.21–0.39]g/L fC1INH 143 [70–130]% C4 0.39 [0.1–0.4] g/L	n.d.
Non14 m	2009	4.4 y	n.d	2.4 y C1INH 0.3 [0.21–0.39]g/L fC1INH 130 [70–130]% C4 0.26 [0.1–0.4]g/L	4.4 y C1INH 0.26 [0.21–0.39]g/L fC1INH 123 [70–130]% C4 0.24 [0.1–0.4]g/L	5.3 y Family mutation ruled out
Non15 f	2009	1 y	**C1INH 0.2 [0.21–0.39]g/L** **C4 0.14 [0.1–0.4]g/L**	1 y C1INH 0.43 [0.21–0.39]g/L fC1INH 168 [70–130]% C4 0.21 [0.1–0.4]g/L		1 y Family mutation ruled out
Non16 m	2010	0.3 y	**C1INH 0.17 [0.21–0.39]g/L** **C4 0.16 [0.1–0.4]g/L**	1.4 y C1INH 0.57 [0.21–0.39]g/L fC1INH 252 [70–130]% C4 0.5 [0.1–0.4]g/L		0.3 y Family mutation ruled out
Non17 m	2013	0 y	Taken for complement measurement, but mishandled. Instead used for genetic testing.			0 y Family mutation ruled out
Non18 f	2014	0 y	n.d.			<0 y Chorionic villous sampling. Family mutation ruled out
Non19 f	2015	0 y	Genetic testing, family mutation ruled out			0 y Family mutation ruled out

Complement values were equivocal (bold font) or normal compared with adult normal range

*N.d*. not determined

**Fig. 1:**
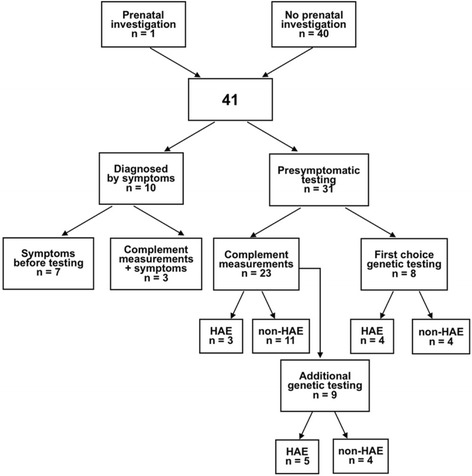
The diagnostic approaches in 41 children investigated for hereditary angioedema

The diagnostic approaches are summed in Fig. 1. Of the children, 7 had symptoms before they were tested. Of these 7 children, 3 had no family history; one had a proven *de novo* mutation, and the parents of the other 2 were asymptomatic with normal C1INH values. Median diagnostic delay in the children with sporadic HAE was 2.8 [2.2–4.8] years versus 1.0 [0.5–1.3] years in cases with a family history. Another 3 children from HAE families were diagnosed at symptom onset based on older blood samples, e.g. their diagnosis was formally established when they had their first attack. Median age of diagnosis among all 7 children diagnosed by symptoms with a family history was 4.4 [2–6.3] years, and for the 3 without family history 8.8 [6.8–13.2] years.

In 31 children, HAE was diagnosed or refuted prior to any symptoms by laboratory testing at mean age 0.4 [0–18.1] years. In 23 of these children, complement values were investigated. In most cases, two complement measurements were performed with at least one occurring after the age of 1 year, but in 7 children HAE was refuted by only one normal complement measurement. Genetic testing was added in 9 other children whose diagnostic course was characterized by repeated complement measurements with incomplete or equivocal results. In 8 children recently investigated, genetic testing was the first choice (at age 0–18.1 years). The mean age of diagnosis or refutation of HAE for all 38 children born into HAE families was 1.5 [0–18.1] years.

Cord blood was used for complement measurements in 9 children and for genetic testing in 4 children. Cord blood complement values were low in 5 children with HAE and equivocal in 4 children (2 with HAE and 2 non-HAE). Results were equivocal in 44% of the complement measurements performed on cord blood compared with 20% (12/60) of the peripheral complement measurements in the cohort.

## Discussion

We investigated clinical features and real-life diagnostic approaches in 41 children from Danish HAE families. Median age at onset was 4 [1–11] years, which is in accordance with a recent Swedish pediatric HAE study [13]. In the other few original studies dedicated to pediatric HAE, age at onset was slightly higher: in a Hungarian study median age at onset was 5 years [14], and in two US studies it was 5.7 and 7 years, respectively [15, 16]. Like Farkas et al., we found that the first attack was most commonly peripheral, closely followed by abdominal attacks (Table 1) [17]. No one had their first attacks in the upper airways. All symptomatic children had experienced abdominal attacks, whereas peripheral and upper airway attacks had occurred in 79% and 29% of the children, respectively. Numbers are similar to those previously reported [16]. Most children had less than one attack per month, which differs from a Hungarian study with 11 of 26 children having frequent attacks [18]. In contrast to a large US study, we found no correlation between age at onset and disease severity (our surrogate measure for disease severity was number of attacks) [19].

Most of the treated children had tried more than one type of therapy, which emphasizes the need for a tailored approach. Unlike other countries [16, 20, 21], androgens were not used in our pediatric cohort. This is noteworthy, because the use of androgens in children with HAE and the potential adverse effects are continuously debated [6, 22]. Home therapy with intravenous C1INH concentrate was established in 9 (64%) of the symptomatic children. Conversely, only 2 of 111 children (1.8%) administered intravenous therapy at home in a UK study, and 28% of treated children were managed at home in Hungary [14, 21]. In our cohort, the youngest child having treatment administered by parents was 5 years old, and the youngest child trained in self-administration was 10 years old. The rate of children able to self-administer was 6 of 9 (67%), which is considerably higher than previously reported [23].

In our cohort, 24% of the children were diagnosed by symptoms and the diagnosis was confirmed by blood testing. This is comparable to 21% in a previous study that also includes children with sporadic HAE [17]. The median diagnostic delay among children with sporadic HAE in our study was 2.8 years compared with a diagnostic delay of 6 years in children without family history in a US study [16].

The mean age at which HAE was diagnosed or refuted was 1.5 years among the 38 children born into HAE families. This is considerably lower than studies including adult patients [1, 19] and also other pediatric studies [13, 15–17]. However, HAE was refuted by only one set of normal complement values in 7 non-HAE children, which must be considered insufficient. Thus, the most recent consensus paper on pediatric HAE specifies that asymptomatic children with a family history of HAE should be considered to have HAE until the diagnosis is ruled out by two complement measurements with at least one of them performed after the age of 1 year [6]. Among children with a family history of HAE, 7 developed symptoms before the diagnosis was formally established with two complement measurements. This is not optimal, as milder HAE symptoms might be missed if children do not have a certified diagnosis.

However, complement measurements do not always give clear results. Complement values can show considerable age-specific variations in children [7, 24–27], and most complement assays do not have reference levels for children. In our cohort, equivocal or incomplete complement results occurred in several cases. This differs from a Spanish HAE study demonstrating a very good agreement between complement values and genetic testing in children [28]. However, only 9 children under the age of 1 year were included. In that study, only C4 was found unreliable, because values were falsely low in 5 non-HAE children. We found, however, 7 cases of C4 values within adult normal range among children with HAE (all without symptoms and untreated when blood samples were taken). As C4 was measured 28 times among children with HAE in our cohort, the sensitivity of low C4 for the diagnosis of HAE was 75% (21/28), which is comparable to a 81% sensitivity previously found [29].

The use of cord blood samples in our cohort seems worth exploring. Among the 9 cord blood complement measurements performed, 4 had equivocal results. In addition, there seemed to be practical challenges of handling blood for the sensitive complement assays in a delivery room as another 3 cord blood samples were mishandled so useful results could not be obtained. To our knowledge, these data form the largest material published on cord blood samples in children from HAE families. A previous study reported data from cord blood in 2 children of HAE patients, of whom one developed HAE [7]. The fluctuating nature of complement values in cord blood has been of concern in international papers [11, 30]. Our findings support that concern and the method is not used anymore at the Danish HAE Centre. Overall, our results sustain the recommendation that complement measurements performed before the age of 1 year should be interpreted with caution [6]

In our experience, some families find it difficult to wait for their child’s HAE diagnosis to be confirmed or refuted by repeated complement measurements. Likewise, physicians are challenged on establishing emergency plans with pediatricians and emergency rooms while the diagnosis is pending. Genetic testing can be helpful in equivocal cases, but until now it has not been recommended as first choice [11]. Moreover, mutations cannot be identified in around five percent of the cases even in clear-cut HAE families [9, 31]. In our cohort, several infants were investigated early by genetic testing, which may partly explain why age at diagnosis was lower in this study. Genetic testing also provided a final answer in some challenging cases and as a consequence it has been our first choice in the most recent cases. It is less sensitive to sample handling compared with complement measurements, and with a known family mutation the genetic analysis has 100% sensitivity and specificity. Also the expense of genetic testing is decreasing. Complement assays are less expensive, but it takes at least two measurements to confirm the diagnosis. Furthermore, there are costs related to having a child under observation for HAE, e.g. supply of emergency medicine. Also the psychological burden of an unresolved case is an important issue.

## Conclusions

We have investigated clinical characteristics of all Danish children with HAE. The age at onset was slightly lower than in other studies, but the distribution of the first attacks was similar to other cohorts. Like in other studies, abdominal and peripheral attacks were the most common and upper airway attacks relatively rare. The rate of home therapy was high and androgens had been avoided.

As the first, we have thoroughly investigated the diagnostic approaches in all children from an entire national HAE cohort, and we present the largest material published on cord blood samples in children from HAE families. Complement values were often equivocal, especially in cord blood samples, and the sensitivity of low C4 for the diagnosis of HAE was only 75%. Consequently, we have changed diagnostic practice and do not use cord blood for complement measurements anymore. Instead, we will advocate for early postnatal genetic testing in children where the family mutation is known.

## Abbreviations

C1INH: C1 inhibitor
C4: Complement factor C4
fC1INH: Functional C1 inhibitor
HAE: Hereditary angioedema
MLPA: Multiplex Ligation-dependent Probe Amplification
